# The VITRO Score (Von Willebrand Factor Antigen/Thrombocyte Ratio) as a New Marker for Clinically Significant Portal Hypertension in Comparison to Other Non-Invasive Parameters of Fibrosis Including ELF Test

**DOI:** 10.1371/journal.pone.0149230

**Published:** 2016-02-19

**Authors:** Stephanie Hametner, Arnulf Ferlitsch, Monika Ferlitsch, Alexandra Etschmaier, Rainer Schöfl, Alexander Ziachehabi, Andreas Maieron

**Affiliations:** 1 Department of Gastroenterology and Hepatology, Division of Internal Medicine IV, Elisabeth Hospital, Linz, Austria; 2 Department of Gastroenterology and Hepatology, Division of Internal Medicine III, Medical University Vienna, Austria; University of Modena & Reggio Emilia, ITALY

## Abstract

**Background:**

Clinically significant portal hypertension (CSPH), defined as hepatic venous pressure gradient (HVPG) ≥10 mmHg, causes major complications. HVPG is not always available, so a non-invasive tool to diagnose CSPH would be useful. VWF-Ag can be used to diagnose. Using the VITRO score (the VWF-Ag/platelet ratio) instead of VWF-Ag itself improves the diagnostic accuracy of detecting cirrhosis/ fibrosis in HCV patients.

**Aim:**

This study tested the diagnostic accuracy of VITRO score detecting CSPH compared to HVPG measurement.

**Methods:**

All patients underwent HVPG testing and were categorised as CSPH or no CSPH. The following patient data were determined: CPS, D’Amico stage, VITRO score, APRI and transient elastography (TE).

**Results:**

The analysis included 236 patients; 170 (72%) were male, and the median age was 57.9 (35.2–76.3; 95% CI). Disease aetiology included ALD (39.4%), HCV (23.4%), NASH (12.3%), other (8.1%) and unknown (11.9%). The CPS showed 140 patients (59.3%) with CPS A; 56 (23.7%) with CPS B; and 18 (7.6%) with CPS C. 136 patients (57.6%) had compensated and 100 (42.4%) had decompensated cirrhosis; 83.9% had HVPG ≥10 mmHg. The VWF-Ag and the VITRO score increased significantly with worsening HVPG categories (P<0.0001). ROC analysis was performed for the detection of CSPH and showed AUC values of 0.92 for TE, 0.86 for VITRO score, 0.79 for VWF-Ag, 0.68 for ELF and 0.62 for APRI.

**Conclusion:**

The VITRO score is an easy way to diagnose CSPH independently of CPS in routine clinical work and may improve the management of patients with cirrhosis.

## Introduction

Portal hypertension (PH) leads to severe complications in patients with cirrhosis. Accordingly, early diagnosis of PH is crucial so that patients can be treated in a timely manner. Adequate treatment helps prevent complications related to PH such as ascites, variceal haemorrhage, hepatic encephalopathy and spontaneous bacterial peritonitis (SBP) and therefore helps reduce the mortality rate.[1, 2]

PH is diagnosed by measuring the hepatic venous pressure gradient (HVPG), and the result is also used for prognostic and therapeutic purposes. Clinically significant portal hypertension (CSPH), defined as HVPG ≥10 mmHg, increases the risk of liver-related mortality and PH-related complications as well as the risk of developing HCC. [1, 3–5] Notably, HVPG ≥12 mmHg elevates the risk of variceal haemorrhage, and a further increase to HVPG ≥20mmHg is associated with poor clinical outcome in patients with cirrhosis. [1, 3, 5] HVPG measurement is thus the “gold standard” for the assessment of PH and correlates with PH-related complications. [6] Measuring HVPG is a minimally-invasive diagnostic procedure that is only available at specialized centres. Accordingly, non-invasive markers for the detection of PH and CSPH are needed to increase diagnostic availability to more patients and consequently to enhance the clinical management of patients with cirrhosis. Although transient elastography (TE) may be a good non-invasive alternative for the diagnosis of PH in patients with cirrhosis, TE is not widely available, it is expensive and, importantly the cut-off values for PH diagnosis have not been established so far. [7–9] Baveno VI consensus conference stated that the introduction of TE allows the early identification of patients at risk developing CSPH. So far liver biopsy, upper gastrointestinal endoscopy showing gastroesophageal varices, HVPG measurement and TE with a cut off ≥ 20 kPa in virus related liver disease have the diagnostic ability to select patients at risk for CSPH and decompensation. Beside TE all these diagnostic tools are invasive but TE is not universal available. Therefore in Baveno VI consensus conference the need of new diagnostic tools, preferably non-invasive tools, should be part of the research agenda. [10]

Von Willebrand factor antigen (VWF-Ag) is released by activated endothelial cells and is therefore an indicator of endothelial cell activation. [11] The endothelium plays a crucial role in many vascular diseases, and endothelial dysfunction is a fundamental component of the increased hepatic vascular tone of cirrhotic livers [12, 13]. VWF-Ag is an established and valuable marker for determining the grade of fibrosis and cirrhosis, for prediction of varices, for PH and for mortality in patients with cirrhosis. [13–17] The AUC for the detection of CSPH using a VWF-Ag cut-off value of ≥241% is 0.85.[16]

Thrombocytopenia is the most common and first hematologic abnormality in patients with cirrhosis due to splenic platelet sequestration, bone marrow suppression and reduced levels or activity of the hematopoietic growth factor thrombopoietin. Moreover thrombocytopenia can be used as a diagnostic criterion for liver cirrhosis. [18, 19] Using the VITRO Score (the **V**on W**i**llebrand factor-Ag/**t**h**ro**mbocyte ratio) rather than the VWF-Ag value itself increases the diagnostic accuracy of determining cirrhosis in patients with AUC values of 0.84–0.89 and determining severe fibrosis in patients with AUC values of 0.79–0.86. [14]

Other non-invasive fibrosis scores are also used to estimate the risk of severe liver fibrosis or cirrhosis. For example, a cut-off value >1.5 for the AST to platelet ratio index (APRI) can be used to detect severe liver fibrosis at an AUC of 0.87. [20] The enhanced liver fibrosis test (ELF test), which consists of a panel of direct biomarkers that includes hyaluronic acid (HA), procollagen III N-terminal propeptide (PIIINP) and tissue inhibitor of metalloproteinase 1 (TIMP-1), can detect liver cirrhosis adequately in most cases at a cut-off value >9.3, and the ELF test has a sensitivity of 93% and a specificity of 86%. [21]

Because VWF-Ag is a valuable marker for both fibrosis and CSPH, we hypothesized that using the VITRO score would improve the diagnostic accuracy of detecting CSPH. We further hypothesized that the ELF test might be able to predict CSPH because of its ability to diagnose fibrosis and cirrhosis. Thus, the aims of our study were (1) to evaluate the diagnostic performance of the VITRO score and ELF test to detect CSPH as defined by HVPG ≥10 mmHg, (2) to compare the diagnostic accuracy of the VITRO score with that of the ELF test, TE, VWF-Ag and APRI and (3) to evaluate whether a combination of non-invasive parameters would improve the diagnostic accuracy of detecting CSPH.

## Patients and Methods

### Patients

This study included patients who underwent HVPG measurement for prognostic, diagnostic or therapeutic reasons and who had an assessment of the VWF-Ag level at the same time at either the Division of Gastroenterology and Hepatology at the Medical University of Vienna or at Elisabethinen Hospital Linz (an academic teaching hospital) between January 2010 and March 2014. Almost every patient underwent HVPG measurement due to cirrhosis that was diagnosed histologically, clinically or by typical radiological findings. Patients with severe cardiopulmonary and/or renal failure, active infections or who showed the presence of pre- or post-hepatic causes of PH were excluded.

The liver disease aetiology, HVPG, medical history (including the presence of ascites, hepatic encephalopathy, oesophageal varices and the stage of cirrhosis according to the Child-Pugh score (CPS)) and the MELD score were recorded for each patient. Patients with no clinical signs of PH and patients with oesophageal varices only were classified as patients with compensated cirrhosis. Patients with a history of ascites and/or variceal bleeding were classified as decompensated according to the D’Amico staging system. The patient age was defined as age at the time of the HVPG measurement. Laboratory parameters, including clinical chemistry and haematological parameters, as well as VWF-Ag, were determined routinely during HVPG measurement, and the VITRO score was calculated. TE to assess liver stiffness was conducted in most patients using FibroScan. The ELF test was performed in a substantial proportion of patients.

This retrospective study was approved by the central ethics committee (Ethikkomission Land OÖ, Linz, Austria; K-49-14 (2.1.12), and the study was conducted in accordance with the tenants of the Declaration of Helsinki. Patients provided written informed consent prior to HVPG measurement and blood test within clinical work up. Patient records were anonymized and de-identified prior to analysis. Written informed consent for analysing patients data and carrying out the study was not requested by the ethics committee because the study was retrospective and there was no additional diagnostic or invasive procedure for any patient due to the study design.

#### VWF-Ag

Plasma levels of VWF-Ag were measured as described previously [22] using a fully automated STA analyser and the VWF-LIA Test (Diagnostic Stargo, Paris, France). The VITRO score was calculated by dividing VWF-Ag by the number of platelets (vWF-Ag/PLT).

#### APRI

APRI was calculated as follows: AST Level (IU/L)/AST upper limit of normal (IU/L)/ platelets (10^9^/L) x 100. [23]

#### HVPG measurement

Portal pressure was evaluated by measuring the HVPG according to international standards as described previously. [24, 25] For each patient, the free and wedged hepatic vein pressure was measured 3 times to calculate the HVPG. Normal portal pressure was defined as an HVPG of 1–5 mmHg, and elevated portal pressure was defined as an HVPG >5–9 mmHg. CSPH was determined by an HVPG ≥10 mmHg.

#### ELF

HA, TIMP-1 and PIIINP were measured by enzyme-linked immunosorbent assay using the ELF test (ADVIA Centaur®; Siemens Healthcare Diagnostics, Inc).

#### TE

Liver stiffness was assessed using TE (FibroScan; Echosens, Paris, France) as described previously. [25] The median liver stiffness value and the interquartile range (IQR) were specified in kPa. The TE results were estimated correctly if the IQR was ≤30% of the median value. [26]

### Statistical analysis

Statistical analyses were performed using SPSS 19.0 (SPSS, Inc., Chicago, IL, USA). Descriptive statistics are reported as median and IQR or percentage. Differences of median values of vWF-Ag, APRI, VITRO score, ELF test and TE between groups with and without CSPH were assessed by Mann-Whitney’s U test. T Test analyses were performed to identify variables that were significantly different between patients with CSPH and patients with HVPG levels below 10 mmHg. Receiver operating characteristic curves (ROCs) were constructed for the assessment of the diagnostic accuracy of vWF-Ag, APRI, VITRO score, ELF test and TE detecting CSPH. Area under the curve (AUC), sensitivity, specificity, positive (PPV) and negative (NPV) predictive values of non-invasive tests were calculated against the gold standard for diagnosis CSPH based on HVPG measurement. PPV was defined as the likelihood of CSPH, NPV was defined as the likelihood of having HVPG levels below 10 mmHg. Furthermore we calculated a logistic regression model. Based on this model we calculated the AUC for the combination of TE and VITRO score. The value with the best sensitivity and specificity in AUC analysis (Youden Index: sensitivity + specificity -1) was chosen as the best cut-off. AUCs were compared using the Hanley McNeil approach. All P-values are two-sided, and P-values <0.05 are considered significant.

## Results

### Patient characteristics

The study included 236 patients with cirrhosis for whom HVPG measurements were available. There were 170 (72%) men and 66 (28%) women with a median age of 57.9 years (IQR: 50–66) in this cohort.

The aetiology of liver cirrhosis was as follows: alcoholic liver disease (ALD) 93 patients (39.4%); chronic hepatitis C (HCV) 67 (28.4%); non-alcoholic steatohepatitis (NASH) 29 (12.3%); other 19 (8.1%); and 28 (11.9%) unknown. A total of 140 patients (59.3%) were classified as CPS A, 56 (23.7%) as CPS B, 18 (7.6%) as CPS C. Due to a lack of clinical knowledge of the severity of either the grade of ascites or hepatic encephalopathy, we were unable to correctly calculate the CPS in 22 (9.3%) individuals. 136 patients (57.6%) had compensated and 100 (42.4%) had decompensated liver disease.

Of the 236 patients, 198 (83.9%) had HVPG ≥10 mmHg and were classified as CSPH; 21 patients (8.9%) had HVPG that was elevated but <10 mmHg and were classified as PH; and 17 patients (7.2%) had HVPG <5 mmHg and were classified as no PH. The patient characteristics are shown in Table 1. Table 2 shows the median values of the patient scores and laboratory parameters (VITRO score, ELF test, TE (kPa), VWF-Ag, platelets, bilirubin, albumin, creatinine, INR, MELD score and APRI) according to CPS, compensation vs. decompensation and PH severity. All parameters shown in Table 2, except creatinine and APRI, differed significantly for the three Child-Pugh stages. Platelets, bilirubin, creatinine and APRI did not differ significantly in compensated versus decompensated patients. Except for bilirubin and creatinine, each parameter differed significantly in patients with CSPH versus patients with HVPG <10 mmHg.

**Table 1:** Demographic characteristics of patients with cirrhosis (n = 236).

*Patient characteristics*	
***Demographic data***	
Age (years)	57,9 (IQR: 50–66)
Male	170 (72%)
***Child Pugh Score***	
CPS A	140 (59,3%)
CPS B	56 (23,7%)
CPS C	18 (7,6%)
***D'Amicco Staging***	
Compensated	136 (57,6%)
Decompensated	100 (42,4%)
***HVPG***	
HVPG≤5 mmHg	17 (7,2%)
HVPG>5 ≤10 mmHg	21 (8,9%)
HVPG≥10 mmHg	198 (83,9%)

**Table 2:** Median values of laboratory parameters and scores according to the Child-Pugh score, D’Amico staging (compensated and decompensated cirrhotic patients) and the extent of portal hypertension.

*parameters*	*Child Pugh Score*				*D'Amicco Staging*			*HVPG*		
	A	B	C	p-value	comp.	decomp.	p-value	< 10mmHg	≥ 10mmHg	p-value
***vWF-Ag***	254	342	395	0,0001	261	329	0,0001	200	306	0,0001
***thrombocytes***	122	148	152	0,026	131	131	n.s.	175	123	0,0001
***VITRO-Score***	2,7	3	4,1	0,48	2,6	3,3	0,011	1,3	3,2	0,0001
***bilirubine***	0,99	2,17	8,5	0,0001	1,2	2,4	n.s.	1,27	2	n.s.
***albumine***	3,9	3,3	2,7	0,0001	3,9	3,3	0,0001	4,1	3,5	0,0001
***creatinine***	0,85	0,9	0,97	n.s.	0,85	0,93	n.s.	0,89	0,88	n.s.
***INR***	1,2	1,4	1,8	0,0001	1,27	1,37	0,028	1,2	1,3	0,0001
***MELD-score***	6,6	10,8	18,1	0,0001	7,7	10	0,001	6,8	9	n.s.
***TE (kPa)***	34	50	50	0,0001	30	49	0,0001	13,4	43,5	0,0001
***ELF-test***	10,5	11,5	12,8	0,001	10,3	11,6	0,0001	9,9	11,1	0,017

Table 1 gives an overview of the most important patient characteristics. Cirrhotic patients are classified by Child Pugh Score (CPS), are grouped between compensated and decompensated cirrhotics and the grade of portal hypertension is evaluated.

Table 2 shows median values of laboratory parameters and scores in patients with cirrhosis according to the Child-Pugh score, D’Amico staging (compensated and decompensated cirrhotic patients) and the extent of portal hypertension as determined by the hepatic venous pressure gradient (HVPG). P-values ≥ 0,05 are not significant (n.s.).

### VWF-Ag and CSPH

**Fig 1:**
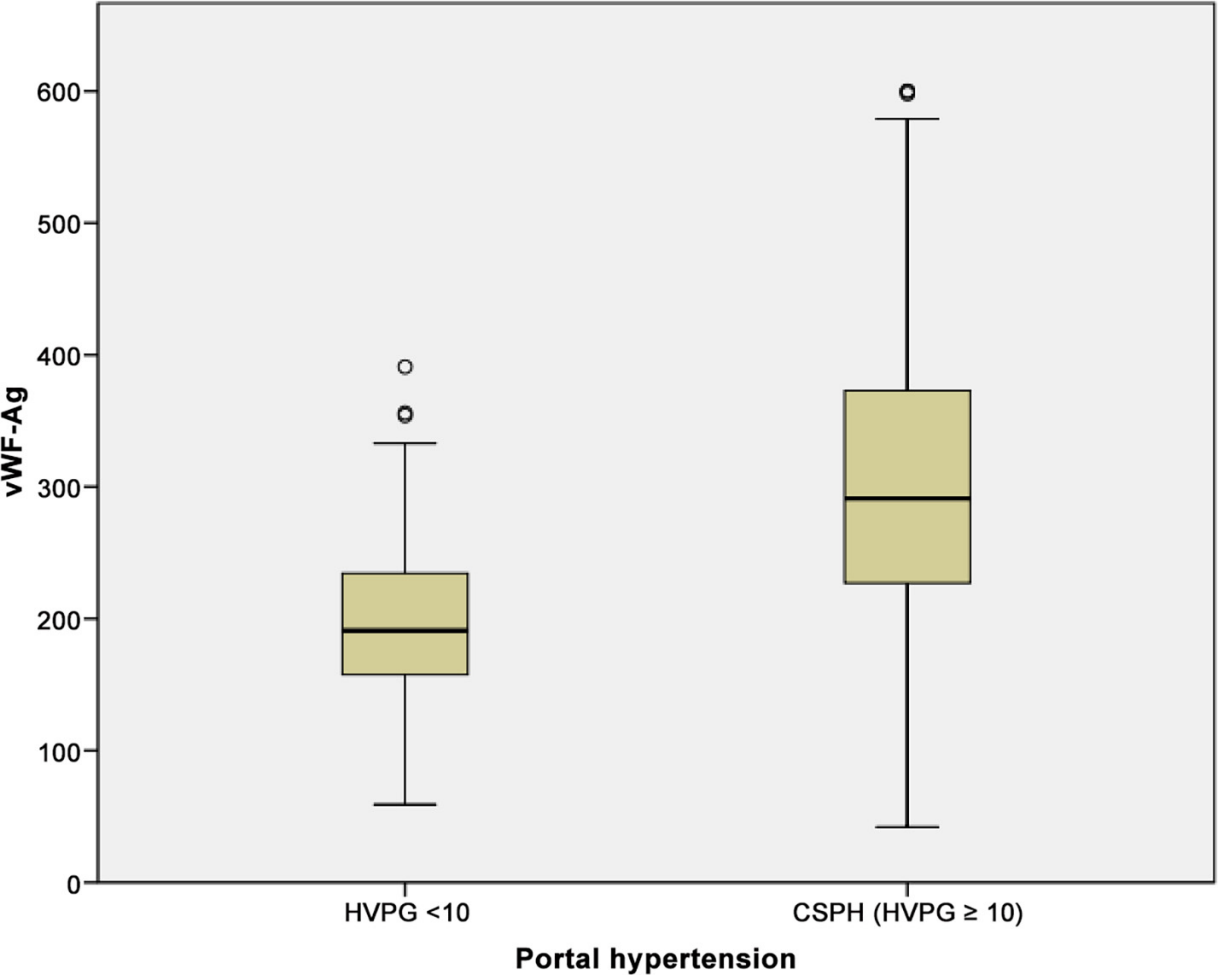
Correlation of Von Willebrand factor antigen (vWF-Ag) and CSPH. Mean values of vWF-Ag shown in boxplots for a cohort of patients with and without CSPH each.

VWF-Ag was significantly higher in patients with CSPH compared to patients with HVPG <10 mmHG (median 306 [IQR 227–373] versus 200 [IQR 157–236]; P<0.0001) as shown in Fig 1. VWF-Ag was higher in patients with oesophageal varices (P<0.018) and ascites (P<0.0001). However, the VWF-Ag level was similar in patients with and without variceal bleeding (P = 0.255). The median VWF-Ag was 261 [IQR 194–306] in compensated patients and 329 [IQR 235–406] in decompensated patients (P<0.0001). There was a significant difference in VWF-Ag between patients with and without CSPH within the CP stages. In CPS A, the median VWF-Ag in non-CSPH was 182 [IQR 155–225] versus 270 [IQR 213–318] in patients with CSPH (P<0.0001). VWF-Ag did not differ significantly in CPS B patients with or without CSPH. In CPS C, all patients had CSPH, with a median VWF-Ag of 395 [IQR 305–501].

### APRI and CSPH

**Fig 2:**
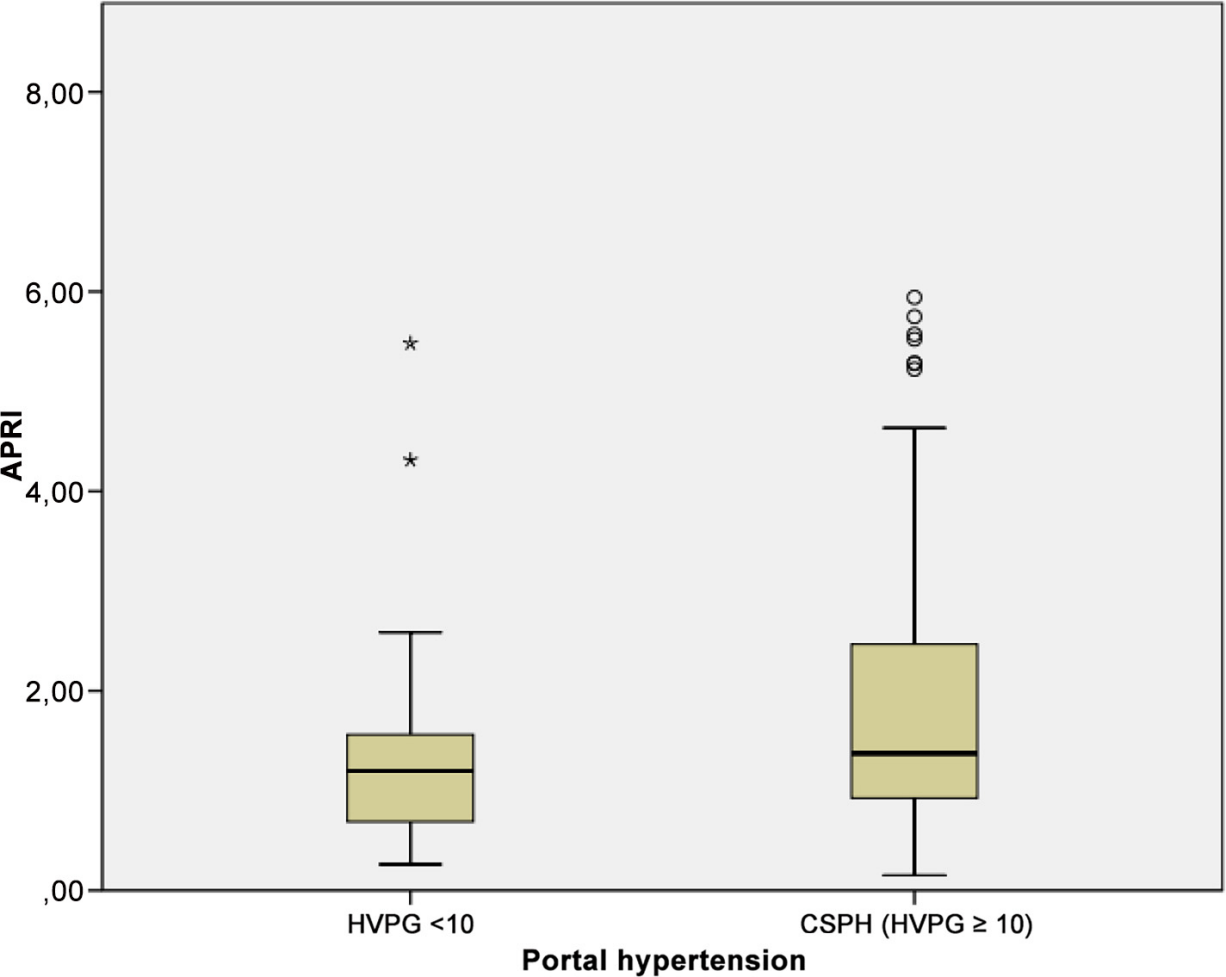
Correlation of aspartate aminotransferase to platelet ratio index (APRI) and CSPH. Mean values of APRI shown in boxplots for a cohort of patients with and without CSPH each.

APRI was significantly higher in patients with CSPH compared to patients with HVPG <10 mmHG (median 2.1 [IQR 0.93–2.47] versus 1.38 [IQR 0.69–1.56]; P = 0.005) as shown in Fig 2. However, the APRI test values were similar between patients with and without oesophageal varices (P = 0.64), ascites (P = 0.24) and variceal bleeding (P = 0.3). The median APRI was 2.01 [IQR 0.97–2.44] in compensated patients and 1.86 [IQR 0.83–2.37] in decompensated patients (P = 0.54). There was no significant difference for APRI between patients with and without CSPH in the three CP stages. In CPS A, the median APRI in non-CSPH was 1.4, and it was 2.2 in patients with CSPH (P = 0.05). APRI did not differ significantly in CPS B patients with or without CSPH (P = 0.9). In CPS C, all patients had CSPH, with a median APRI of 1.9.

### VITRO score and CSPH

**Fig 3:**
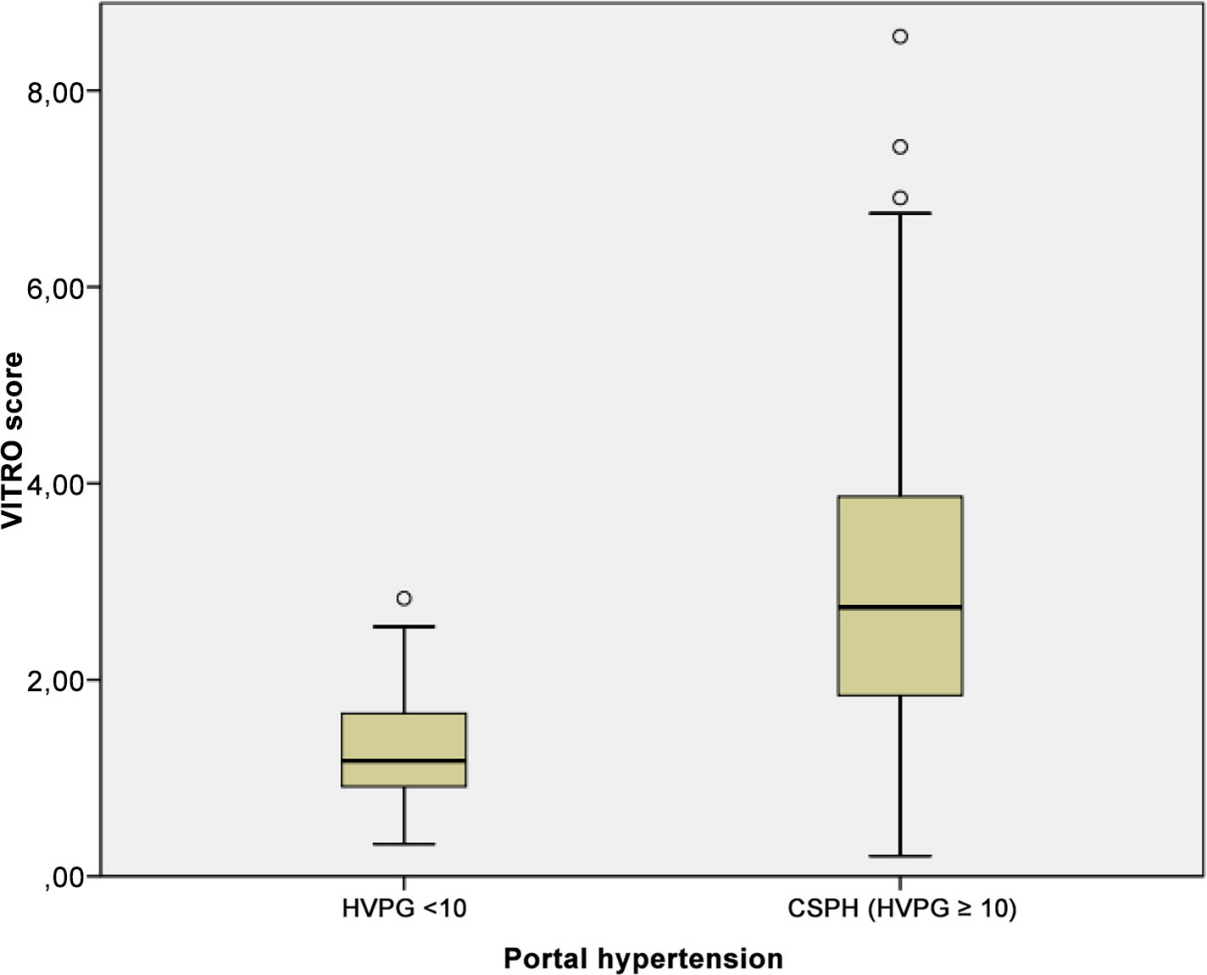
Correlation of VITRO (VWF-Ag/platelets) score and CSPH. Mean values of VITRO score shown in boxplots for a cohort of patients with and without CSPH each.

The VITRO score was significantly higher in patients with CSPH compared to patients with HVPG <10 mmHG (median 3.21 [IQR 1.84–3.87] versus 1.29 [IQR 0.91–1.66]; P<0.0001) as shown in Fig 3. The VITRO score was higher in patients with oesophageal varices (P<0.0001) and ascites (P<0.014), but did not differ significantly in patients with variceal bleeding (P<0.06). The median VITRO score was 2.6 [IQR1.5–3.3] in compensated patients and 3.3 [IQR 1.8–4.2] in decompensated patients (P<0.014). There was a significant difference in the mean VITRO scores in patients with and without CSPH within the CP stages. In CPS A, the median VITRO score in non-CSPH was 1.2 [IQR 0.7–1.4], while it was 3 [IQR 1.8–3.6] in patients with CSPH (P<0.0001). In CPS B patients, the median VITRO score was 1.6 [IQR 1.2–2] in non-CSPH patients and 3.1 [IQR 1.6–4.5] in patients with CSPH (P = 0.14). In CPS C, all patients had CSPH, and the median VITRO score was 4.1 [IQR 1.7–4.6].

### ELF test and CSPH

**Fig 4:**
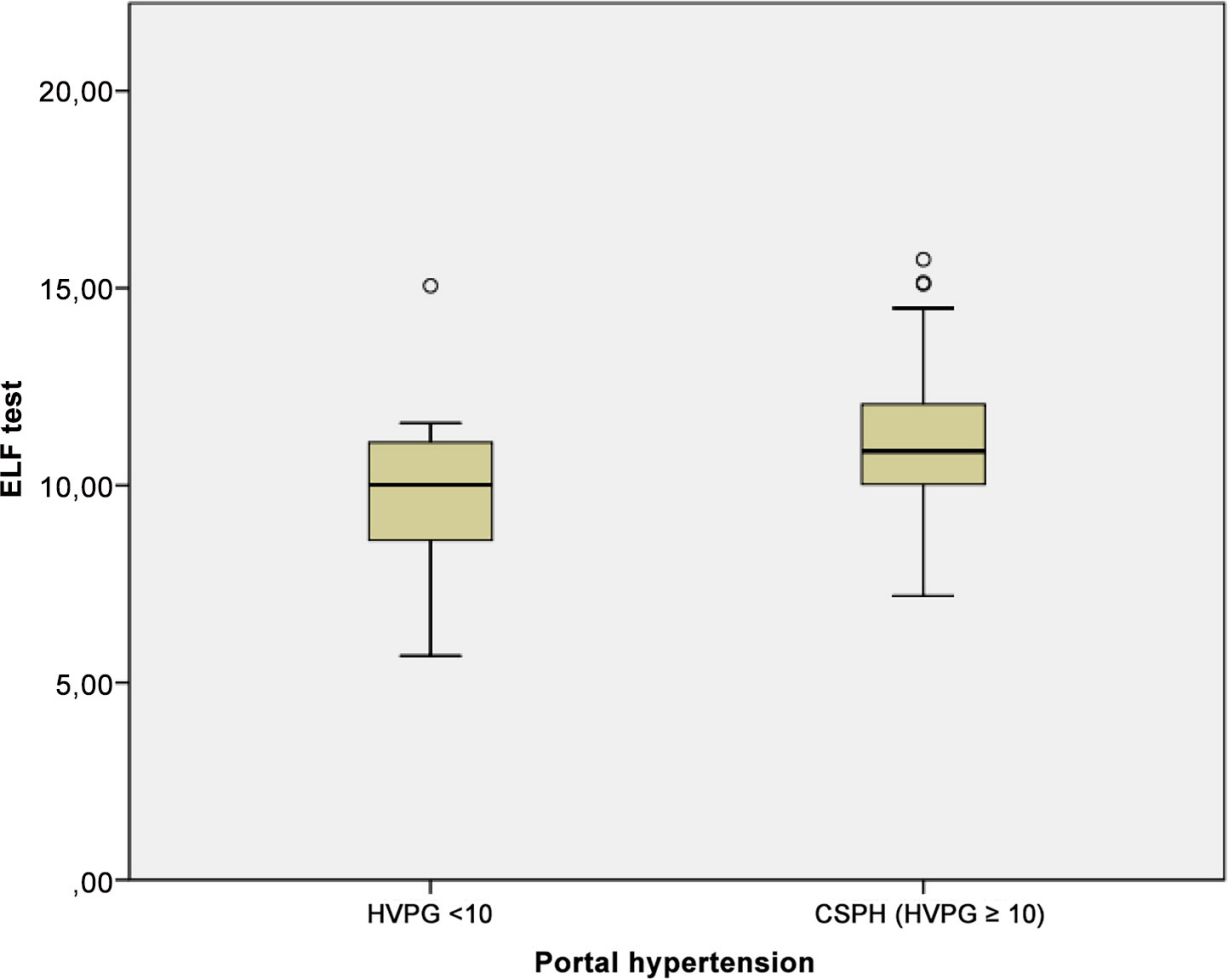
Correlation of enhanced liver fibrosis test (ELF) test and CSPH. Mean values of ELF test shown in boxplots for a cohort of patients with and without CSPH each.

ELF was significantly higher in patients with CSPH compared to patients with HVPG <10 mmHG (median 11.1 [IQR 10–12] versus 9.9 [IQR 8.6–11.1]; P<0.037), Fig 4. However, ELF was similar in patients with and without oesophageal varices and in those with and without a history of bleeding (P = 0.35 and P = 0.95, respectively). The ELF was significantly higher in patients with ascites (P<0.0001). The median ELF was 10.3 [IQR8.9–11.4] in compensated patients and 11.6 [IQR 10.3–12.6] in decompensated patients (P<0.0001). There was no significant difference in ELF between patients with and without CSPH within the CP stages. In CPS A, the median ELF in non-CSPH was 10 [IQR 8.9–11.1], and it was 10.6 [IQR 9.3–11.7] in patients with CSPH (P = 0.4). Similarly, in CPS B patients, the median ELF was 9.7 [IQR 8–11.4] in non-CSPH patients and 11.7 [IQR 10.4–12.6] in patients with CSPH (P = 0.45). In CPS C, all patients had CSPH, and the median ELF was 12.7 [IQR 11.7–13.9].

### TE and CSPH

**Fig 5:**
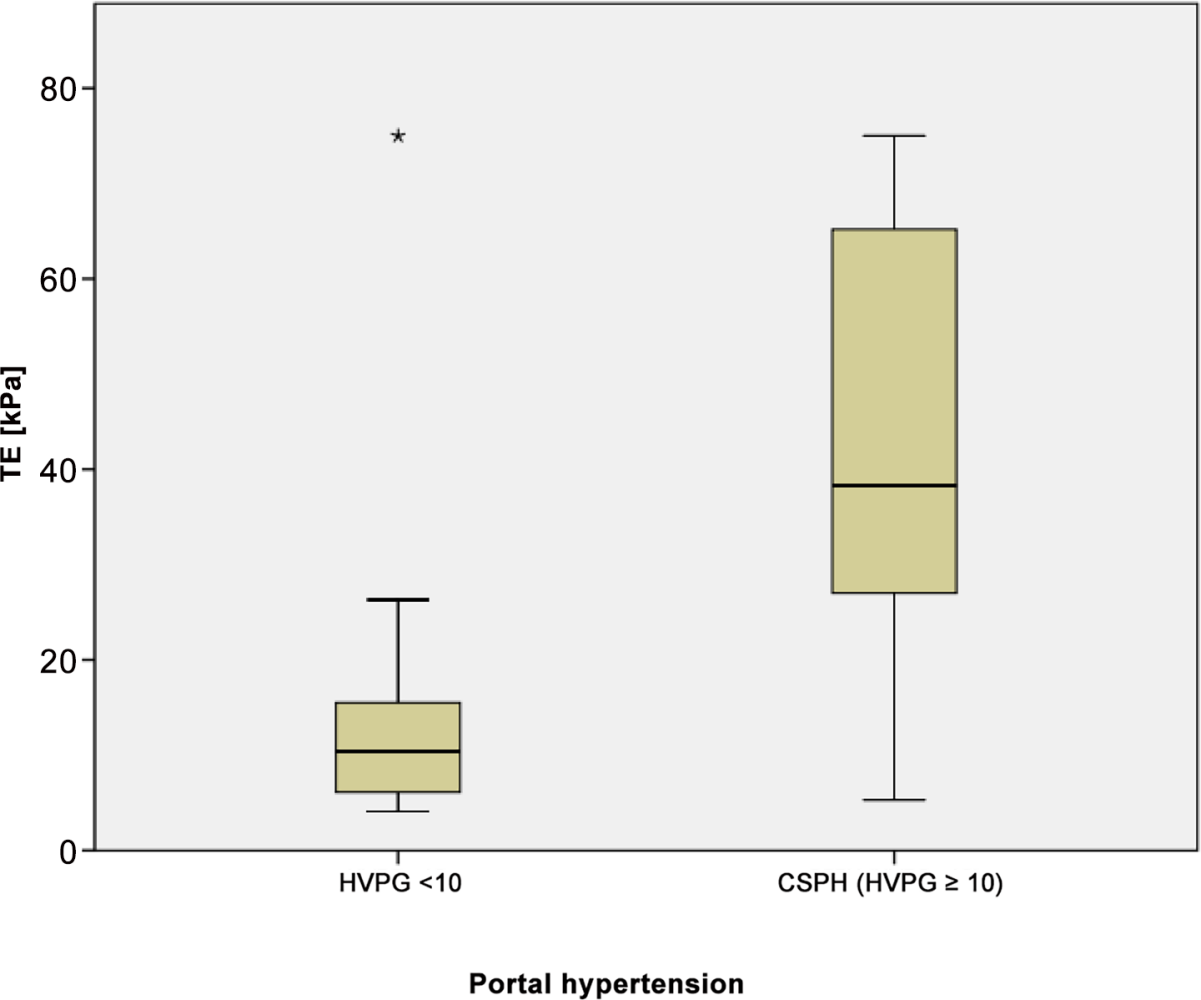
Correlation of transient elastography (TE; kPa) and CSPH. Mean values of TE shown in boxplots for a cohort of patients with and without CSPH each.

TE was significantly higher in patients with CSPH compared to patients with HVPG <10 mmHG (median 43 kPa [IQR 27–65] versus 13 kPa [IQR 6–16]; P<0.0001), Fig 5. TE was higher in patients with oesophageal varices (P<0.001) and ascites (P<0.0001). There was no significant correlation between TE and variceal bleeding (P = 0.35). The median TE was 30 kPa [IQR12–43] in compensated patients and 49 kPa [IQR 33–75] in decompensated patients (P<0.0001). TE varied significantly in CPS A patients with CSPH, 10.9 kPa [IQR 6–15], compared to patients without CSPH 40 kPa [IQR 25–53] (P<0.0001). The TE did not differ significantly in CPS B patients with or without CSPH.

### Diagnostic accuracy of VWF-Ag, APRI, VITRO score, ELF and TE in the detection of CSPH

**Fig 6:**
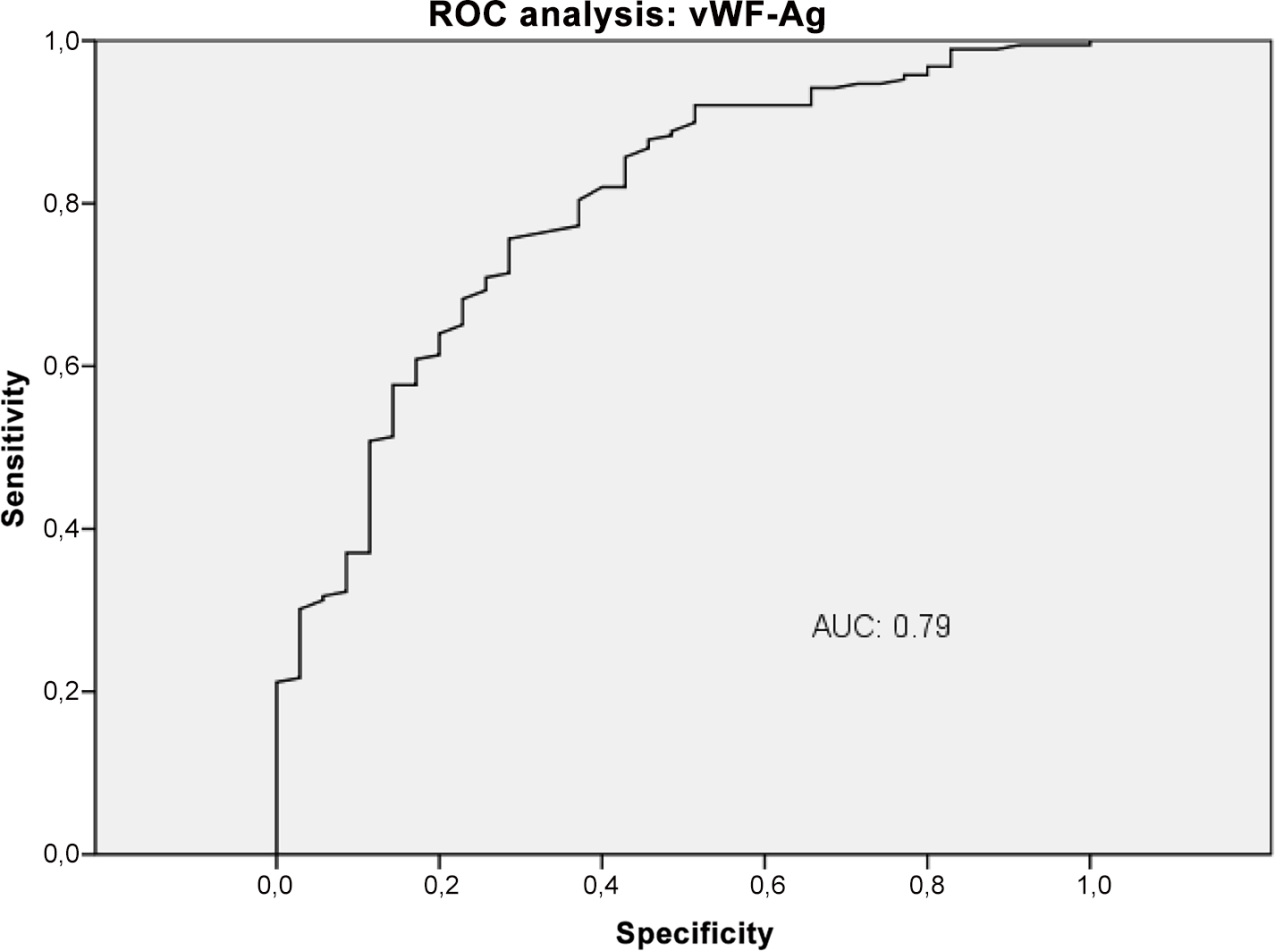
ROC analysis shows the ability of Von Willebrand factor antigen (vWF-Ag) predicting clinically significant portal hypertension (CSPH). ROC analysis showing the diagnostic ability of vWF-Ag detecting CSPH with an AUC of 0.79.

**Fig 7:**
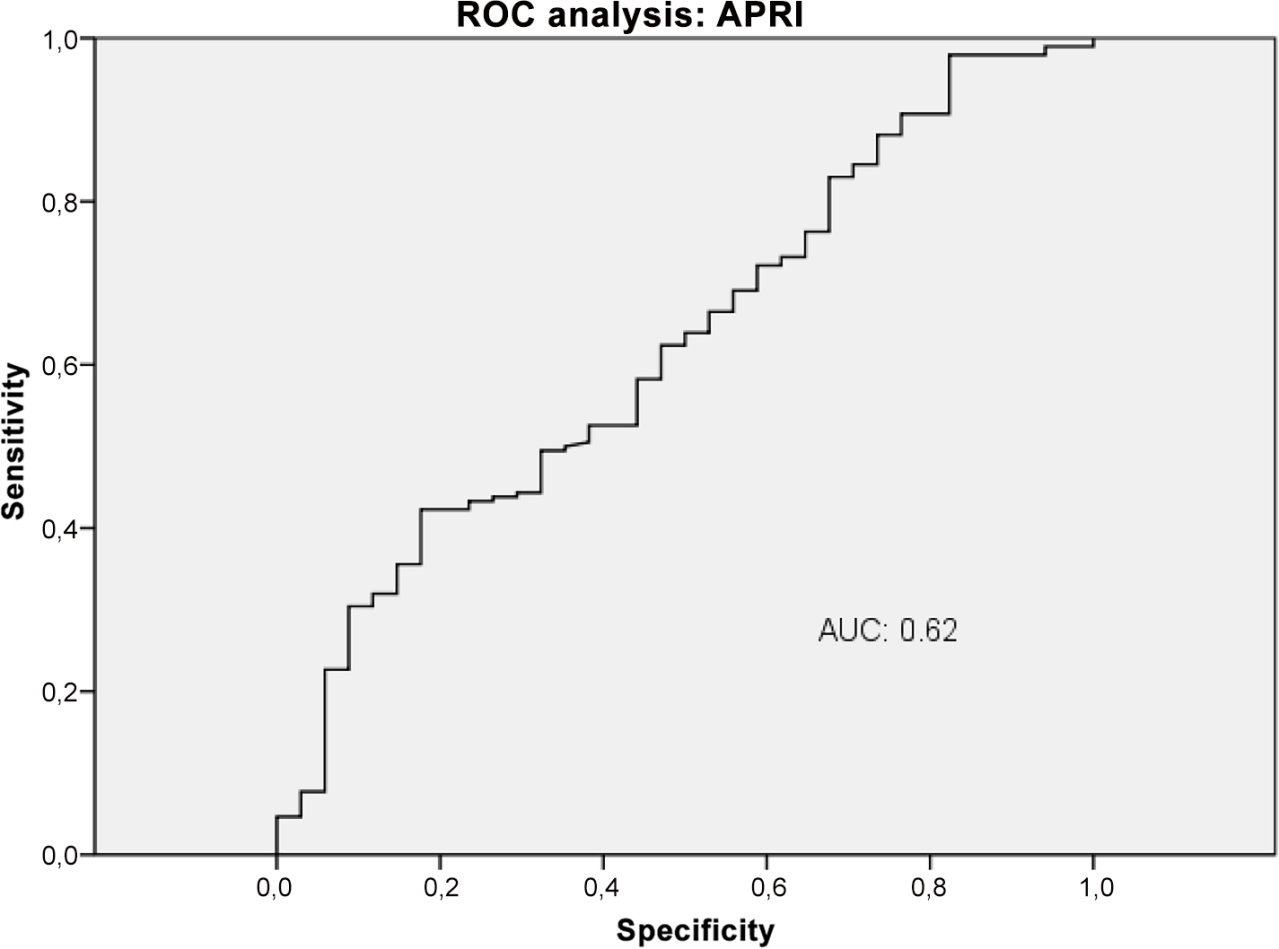
ROC analysis shows the ability of aspartate aminotransferase to platelet ratio index (APRI) predicting clinically significant portal hypertension (CSPH). ROC analysis showing the diagnostic ability of APRI detecting CSPH with an AUC of 0.62.

**Fig 8:**
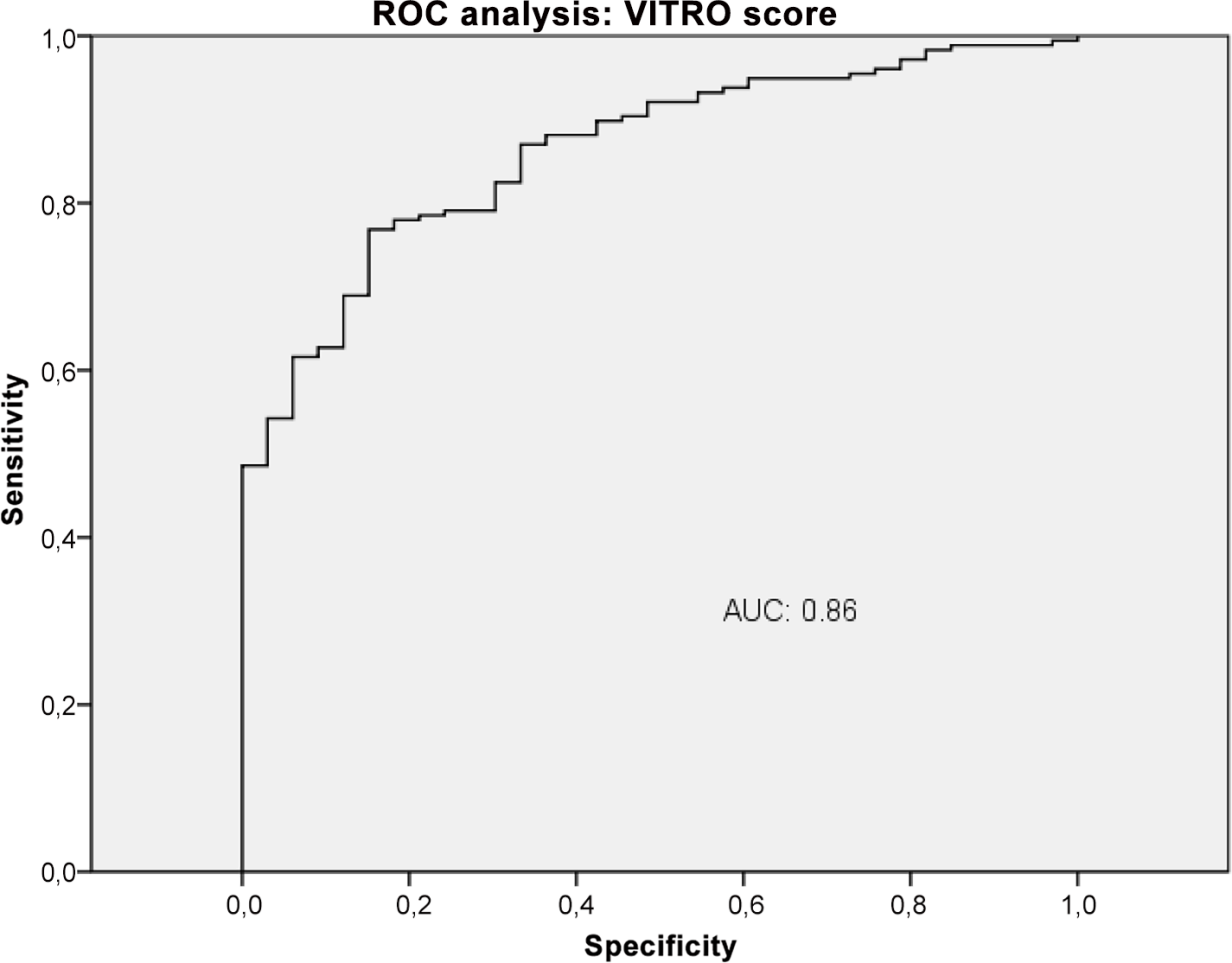
ROC analysis shows the ability of VITRO (VWF-Ag/platelets) score predicting clinically significant portal hypertension (CSPH). ROC analysis showing the diagnostic ability of VITRO score detecting CSPH with an AUC of 0.86.

**Fig 9:**
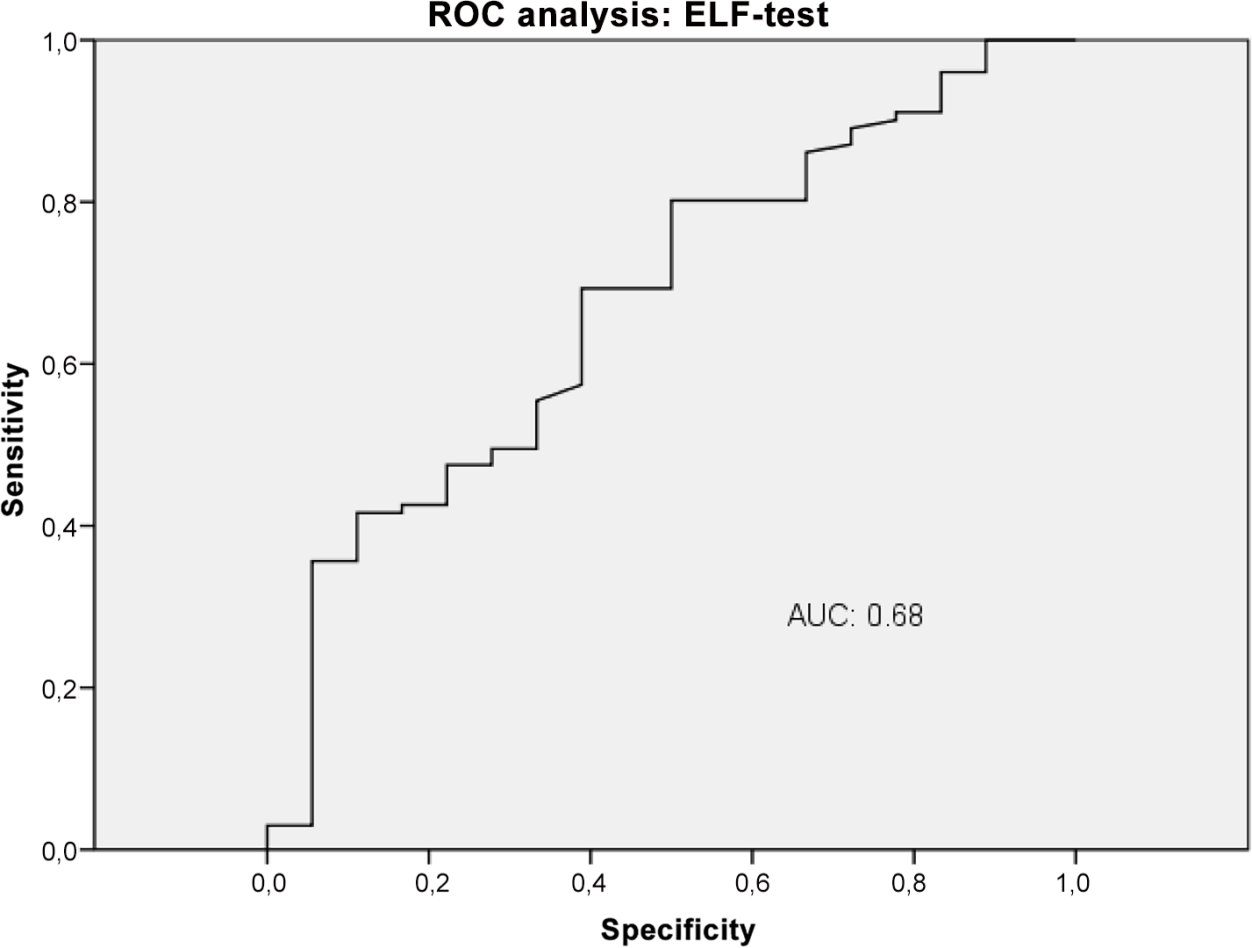
ROC analysis shows the ability of enhanced liver fibrosis test (ELF) predicting clinically significant portal hypertension (CSPH). ROC analysis showing the diagnostic ability of ELF test detecting CSPH with an AUC of 0.68.

**Fig 10:**
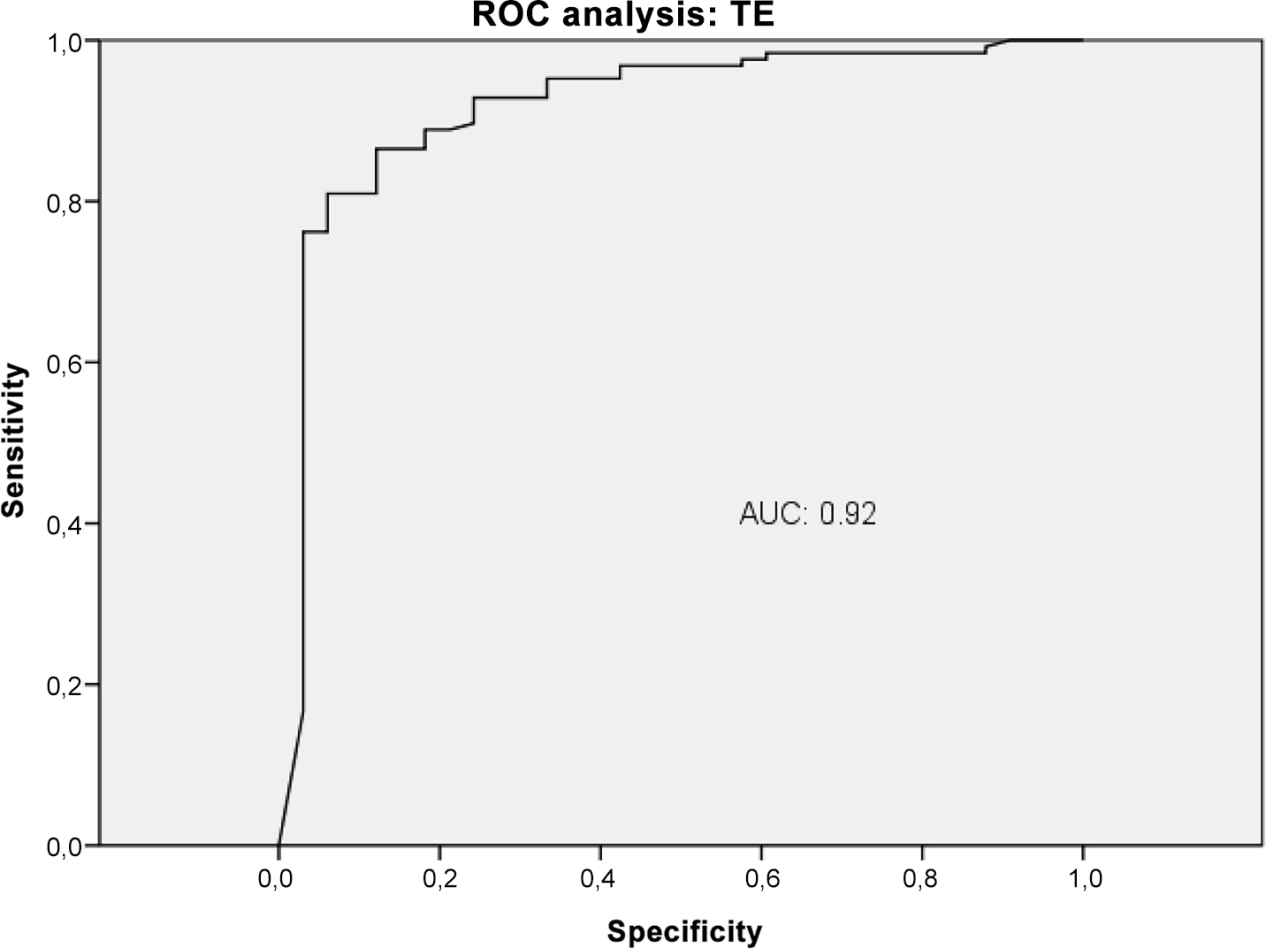
ROC analysis shows the ability of transient elastography (TE) predicting clinically significant portal hypertension (CSPH). ROC analysis showing the diagnostic ability of TE detecting CSPH with an AUC of 0.92.

The diagnostic performance of VWF-Ag, APRI, VITRO score, ELF and TE for predicting CSPH was analysed by AUROC. VWF-Ag had an AUC of 0.79 (CI 0.72–0.87), a sensitivity of 76% and a specificity of 71% at a cut-off >226 (Fig 6). APRI showed an AUC of 0.62 (CI 0.53–0.72), a sensitivity of 42% and a specificity of 82% at a cut-off >1.74 (Fig 7). The VITRO score showed an AUC of 0.86 (CI 0.81–0.91), a sensitivity of 80% and a specificity of 70% at a cut-off >1.58 in predicting CSPH (Fig 8) with a PPV of 93.2 and a NPV of 40.1. ELF showed an AUC of 0.68 (CI 0.59–0.76), a sensitivity of 42% and a specificity of 89% at a cut-off >11.4 in predicting CSPH (Fig 9). TE performed the best, with an AUC of 0.92 (CI 0.86–0.96), a sensitivity of 81% and a specificity of 93% at a cut-off >24.8 in predicting CSPH (Fig 10) with a PPV of 98.4 and a NPV of 48.4. In our cohort, 44.9% patients had correct results using TE, 22.5% did not have a correct result due to high IQR and 32.6% did not undergo TE because it was not available or there was a technical issue.

**Fig 11:**
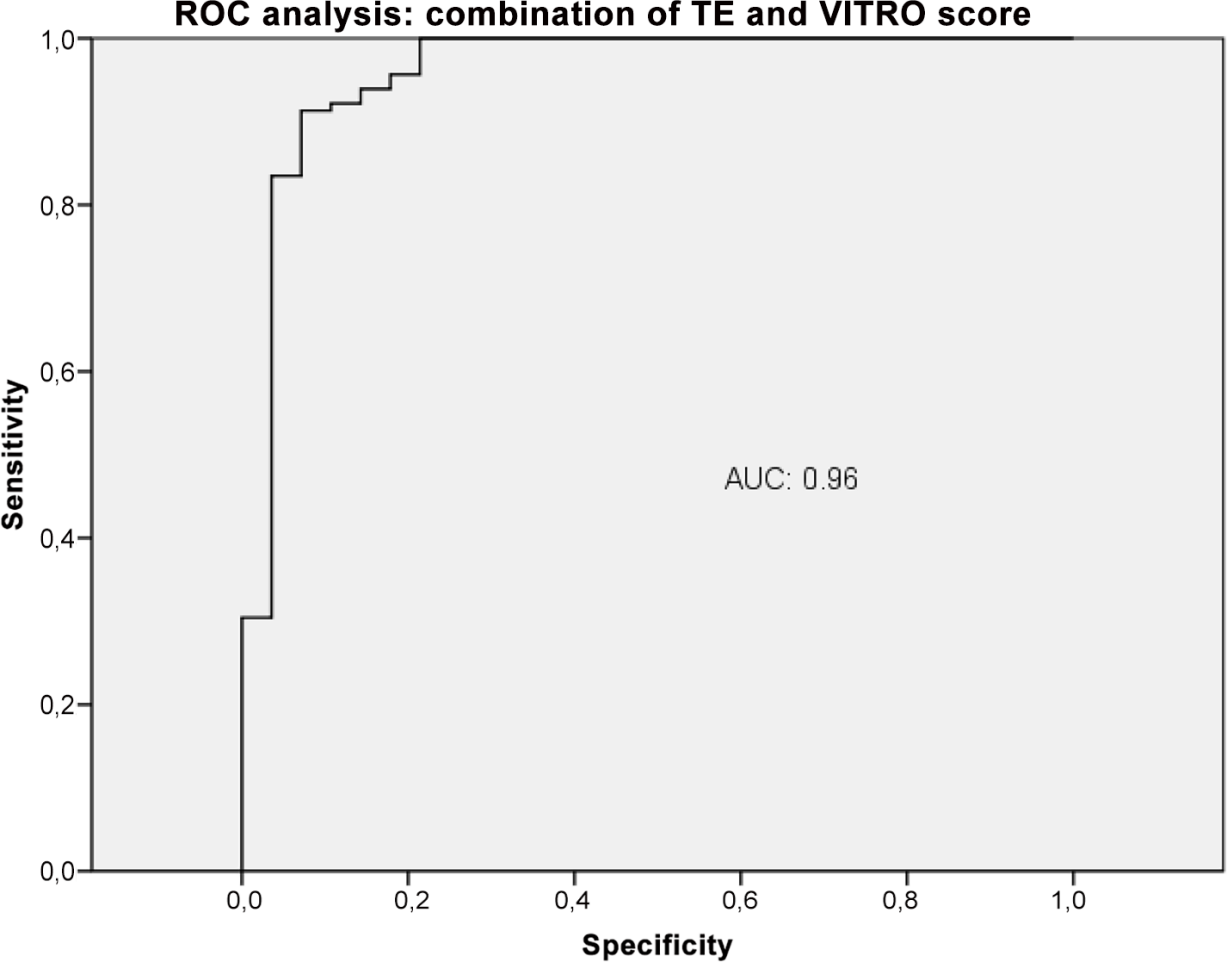
ROC analysis shows the ability of the combination of TE and VITRO score predicting clinically significant portal hypertension (CSPH). ROC analysis showing the diagnostic ability of TE in combination with VITRO score detecting CSPH with an AUC of 0.96.

The VITRO score substantially improved detection of CSPH compared to VWF-Ag alone, with an AUC of 0.79 (CI 0.7–0.85), a sensitivity of 80% and a specificity of 63% at a cut-off >218. The diagnostic accuracy of TE for detecting CSPH was significantly better than the VITRO score (P<0.03). The VITRO score was second best for correctly predicting CSPH. Both the TE and VITRO score performed well in HCV patients, predicting CSPH with AUCs of 0.97 and 0.91 (P = 0.22); in the entire cohort, the AUCs were 0.92 and 0.86, respectively. The combination of TE and VITRO score showed an improvement of the diagnostic ability detecting CSPH with an AUC of 0.96 (CI 0.91–0.98), a sensitivity of 91% and a specificity of 93% at a cut-off >0.71 (Fig 11) with a PPV of 98.5 and a NPV of 67.2. The diagnostic accuracy of the combination of TE and VITRO score was significantly better than VITRO score alone (P = 0.001) but did not differ significantly compared to TE alone (P = 0.6). An overview of the diagnostic accuracy of non-invasive biomarkers detecting CSPH is given (Table in S1 File). The combination of other non-invasive biomarkers did not show further improvement of the diagnostic ability detecting CSPH.

Additionally we calculated whether non-invasive biomarkers could help diagnosing HVPG ≥ 12mmHg which would have greater clinical significance for bleeding risk. The AUCs for diagnosing HVPG ≥ 12 mmHg showed a slightly worse diagnostic ability compared to the AUCs of diagnosing CSPH (HVPG ≥ 10 mmHg) (Table in S2 File).

## Discussion

This study clearly demonstrates that the VITRO score has diagnostic and predictive value as a novel, non-invasive marker in patients with PH due to cirrhosis. PH severity was assessed using the gold standard method (HVPG measurement) for each patient. We showed a clear correlation between CSPH and the VITRO score as well as between CSPH and other non-invasive tests, such as the ELF test and TE. Moreover, we showed that the VITRO score might be a valuable, non-invasive marker for risk stratification in patients with CPS A and B cirrhosis. The assessment of VITRO score could help to diagnose patients with CSPH easily and get them on diagnostic and therapeutic track according to Baveno VI guidelines.

An adequate explanation on the pathophysiological mechanisms of elevated vWF-Ag in our patients with cirrhosis cannot be given by our data. Platelets themselves and thrombotic risk factors in patients with viral hepatitis are associated with higher fibrosis stages and might be a promotor for liver injury themselves. The increased shear stress or bacterial infection may lead to elevated vWF-Ag levels in cirrhosis. Additionally there might be induction of the synthesis of vWF-Ag in the cirrhotic liver. Increased activity of ADAMTS13 (vWF cleaving protease) might lead to a reduced clearance of vWF-Ag in patients with CSPH. [27–29]

The clinical consequences of cirrhosis are usually related more to CSPH than to any other cause. Although HVPG measurement is the gold standard for investigating the degree of PH, it is not in common use because it is minimally invasive, it is not widely available, it is expensive and it is not successful for technical reasons in up to 4% of patients. [30] Its invasiveness plus the lack of general availability of HVPG measurement hinders the broad use of pressure-guided diagnostic and therapeutic algorithms in patients with cirrhosis. To better prevent PH-related complications, new staging systems are needed for patients with cirrhosis. VWF-Ag levels can be determined easily and cheaply using standard laboratory techniques, making it easy to determine the VWF-Ag/platelets ratio (i.e. the VITRO score). Moreover it has been shown that vWF-Ag levels add information on mortality on top of MELD score. Therefore one could argue that introducing vWF-Ag and VITRO score on the one hand can diagnose CSPH and on the other hand it might be used as a prognostic tool. VITRO score performs well in patients with CSPH, however it might be difficult to identify patients who have HVPG values just above 10 mmHg with non-invasive parameters. Therefore in these cases we believe that HVPG measurement will remain the standard of care even if non-invasive parameters such as VITRO score, vWF-Ag and other non-invasive scores are established. However, there are known limitations to using VWF-Ag levels in patients with cirrhosis. On the one hand, infections, malignancies, physical training or interferon-based therapy can elevate the VWF-Ag levels. [31, 32] On the other hand, acute bleeding or hereditary VWF-Ag deficiency can lower VWF-Ag levels, leading to underestimation of the degree of PH. Our VWF-Ag cut-off for predicting CSPH was 218%, which is in line with the cut-off of 215% found by Ferlitsch et al. and the cut-off of 216% published by La Mura. [16, 17]

The VITRO score showed a clear correlation with CSPH (P<0.0001). It also correlated with clinical manifestations of PH, such as oesophageal varices (P<0.004), ascites (P<0.014), bleeding (P<0.01) and decompensation (P<0.011). Similar results were shown previously by La Mura et al. and Ferlitsch et al. using VWF-Ag instead of the VITRO score. [16, 17] Maieron et al. showed that the VITRO score improves diagnostic accuracy in the prediction of cirrhosis compared to VWF-Ag alone. [14] We found the same diagnostic improvement in predicting CSPH using the VITRO score instead of VWF-Ag alone. Furthermore, in our large cohort, we demonstrated an impressive correlation between CSPH and the VITRO score, which is independent of CPS. Therefore, the VITRO score can be used for risk stratification in patients with CPS A and B cirrhosis. [11, 16] The diagnostic accuracy of the VITRO score for detecting CSPH shows an AUC of 0.86 (CI 0.81–0.91) with a sensitivity of 80% and a specificity of 70% at a cut-off >1.58. It is remarkable that a cheap and easy to obtain laboratory parameter shows AUROCs for the diagnosis of CSPH that are comparable to those of other non-invasive tests.

APRI, a well-established non-invasive fibrosis marker, has a median value >1.5 for patients with cirrhosis. [23] However, APRI does not provide additional information in the detection of CSPH. The ELF test, another non-invasive test that was designed to evaluate fibrosis stage [33], is worse than the VITRO score and TE in terms of detecting CSPH. ELF increases significantly in decompensated patients and in patients with CSPH, but it is not adequate for detecting clinical complications of CSPH such as oesophageal varices and bleeding nor is it useful in risk stratification of patients with CPS A and B cirrhosis. Therefore, although ELF is a valuable non-invasive marker for the detection of liver cirrhosis [34], it is insufficient for diagnosing CSPH.

TE is similar to the VITRO score for identifying clinical manifestations of PH such as ascites and oesophageal varices, decompensated patients and for determining risk stratification in CPS A patients. TE is even better for detecting CSPH, with an AUC of 0.92 (CI 0.86–0.96), a sensitivity of 81% and a specificity of 93% at a cut-off >24.8. Moreover a combination of TE and VITRO score shows additional improvement of detecting CSPH with an AUC of 0.96 (CI 0.91–0.98). However, 25 patients with CSPH showed liver stiffness <25 kPa. In daily clinical practice, TE is only accurate in up to two-thirds of patients, plus the TE system and its maintenance are associated with substantial costs. [35] Moreover, in our cohort, adequate results were obtained in less than half of the patients. The literature notes that there is a percentage of up to 40% of patients in which TE results are inadequate due to high IQR or inadequate measurement. [26, 35] In our large cohort, one weakness of TE that it was not available to about one-third of the patients due to technical issues or to operator shortage. Moreover, in 22% of the patients, the TE results were not reliable. Therefore, even though TE shows the best prediction for CSPH, it seems that there is great need of improvement as a routing diagnostic tool since half of the patients fail to obtain an adequate result. In fact, one limitation of this study is that we did not have TE data for 32.6% of the patients.

In conclusion, the VITRO score offers an easy way to diagnose CSPH independently of CPS in routine clinical work and may therefore improve the management of patients with cirrhosis.

## Supporting Information

S1 File This file contains Table A: Overview of different non-invasive markers/scores detecting CSPH. (DOCX)

S2 File This file contains Table B: Overview of different non-invasive markers/scores detecting HVPG ≥ 12mmHg. (DOCX)
