# Supplementary material for: The VITRO Score (Von Willebrand Factor Antigen/Thrombocyte Ratio) as a New Marker for Clinically Significant Portal Hypertension in Comparison to Other Non-Invasive Parameters of Fibrosis Including ELF Test
Source: PLoS One. 2016 Feb 19;11(2):e0149230. doi: 10.1371/journal.pone.0149230 (PMC4760704; doi:10.1371/journal.pone.0149230)
Supplement: S1 File — (DOCX) [file pone.0149230.s001.docx]

**Supporting Information**

**S1**

The VITRO score (Von Willebrand factor antigen/thrombocyte ratio) as a new marker for clinically significant portal hypertension in comparison to other non-invasive parameters of fibrosis including ELF test

Stephanie Hametner, Arnulf Ferlitsch, Monika Ferlitsch, Alexandra Etschmaier, Rainer Schöfl, Alexander Ziachehabi, Andreas Maieron

**Table of contents**

Supporting Table S1 2

**Supplementary Table**

Supplementary Table A. Overview of different non-invasive markers/scores detecting CSPH

| detection of CSPH (HVPG ≥ 10 mmHg) | | | | | |
| --- | --- | --- | --- | --- | --- |
| scores | AUC | CI | cut off | sensitivity | specificity |
| vWF-Ag | 0.79 | 0.71-0,87 | > 226 | 75.6 | 71.4 |
| APRI | 0.62 | 0.53-0.72 | > 1.74 | 42 | 82 |
| VITRO score | 0.86 | 0.81-0.91 | > 1.58 | 80 | 70 |
| ELF test | 0.68 | 0.59-0.76 | > 11.4 | 42 | 89 |
| TE | 0.92 | 0.86-0.96 | > 24.8 | 81 | 93 |
| TE + VITRO | 0.96 | 0.91-0.98 | > 0.7 | 91 | 93 |

CSPH, clinically significant portal hypertension; HVPG, hepatic venous pressure gradient; AUC, area under the curve; CI, confidence interval;
