# Supplementary material for: The VITRO Score (Von Willebrand Factor Antigen/Thrombocyte Ratio) as a New Marker for Clinically Significant Portal Hypertension in Comparison to Other Non-Invasive Parameters of Fibrosis Including ELF Test
Source: PLoS One. 2016 Feb 19;11(2):e0149230. doi: 10.1371/journal.pone.0149230 (PMC4760704; doi:10.1371/journal.pone.0149230)
Supplement: S2 File — (DOCX) [file pone.0149230.s002.docx]

**Supporting Information**

**S2**

The VITRO score (Von Willebrand factor antigen/thrombocyte ratio) as a new marker for clinically significant portal hypertension in comparison to other non-invasive parameters of fibrosis including ELF test

Stephanie Hametner, Arnulf Ferlitsch, Monika Ferlitsch, Alexandra Etschmaier, Rainer Schöfl, Alexander Ziachehabi, Andreas Maieron

**Table of contents**

Supporting Table S2 2

**Supplementary Table**

Supplementary Table B. Overview of different non-invasive markers/scores detecting HVPG ≥ 12mmHg

| detection of HVPG ≥ 12 mmHg | | | | | |
| --- | --- | --- | --- | --- | --- |
| scores | AUC | CI | cut off | sensitivity | specificity |
| vWF-Ag | 0.79 | 0.74-0.85 | > 226 | 78 | 68 |
| APRI | 0.55 | 0.49-0.6 | > 2.1 | 34 | 80 |
| VITRO score | 0.78 | 0.71-0.83 | > 1.77 | 77 | 72 |
| ELF test | 0.67 | 0.6-0.75 | > 11.4 | 43 | 83 |
| TE | 0.9 | 0.85-0.94 | > 24.8 | 83 | 85 |
| TE + VITRO | 0.91 | 0.83-0.98 | > 0.76 | 91 | 83 |

HVPG, hepatic venous pressure gradient; AUC, area under the curve; CI, confidence interval;
